# Supplementary material for: Lower Functional and Proportional Characteristics of Cord Blood Treg of Male Newborns Compared with Female Newborns
Source: Biomedicines. 2021 Feb 9;9(2):170. doi: 10.3390/biomedicines9020170 (PMC7915235; doi:10.3390/biomedicines9020170)
Supplement: Supplementary file 1 [file biomedicines-09-00170-s001.pdf]

Supplementary figure 1

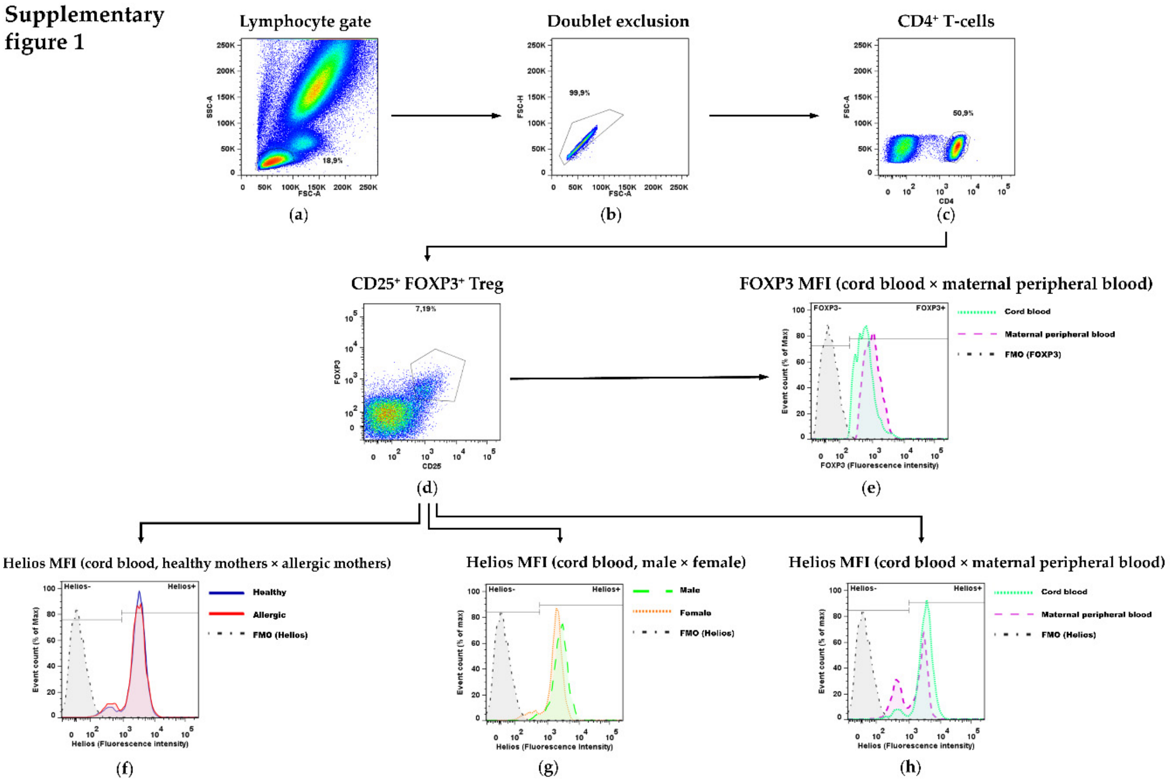

Supplementary figure 1. Gating strategy used for total Treg, iTreg and nTreg proportion determination.

(**a**-**d)** The gating strategy used to identify CD25⁺FOXP3⁺ Treg. (**a**) Lymphocyte gate was set out of all events according to the size (FSC-A) and complexity (SSC-A). (**b**) Doublet exclusion was done based on FSC-A × FSC-H discrimination. (**c**) CD4⁺ T-cells were selected from singlet lymphocytes. (**d**) Treg were identified as CD25⁺FOXP3⁺ cells within the CD4⁺ T-cell population. (**e**) Median of fluorescence intensity (MFI) of FOXP3 in CD25⁺FOXP3⁺ Treg was quantified. Representative histograms for cord blood samples and maternal peripheral blood samples as well as a histogram of fluorescence minus one (FMO) for FOXP3 are shown. (**f**-**h**) CD25⁺FOXP3⁺ Treg were divided into Helios⁻ (induced Treg, iTreg) and Helios⁺ (natural Treg, nTreg) subpopulations, MFI of Helios in CD25⁺FOXP3⁺ Treg was quantified. Representative histograms and FMO control for Helios are shown. (**f**) Representative histograms of Helios in children of healthy and allergic mothers. (**g**) Representative histograms of Helios in male and female children. (**h**) Representative histograms of Helios in cord blood and maternal peripheral blood samples.

Supplementary figure 2

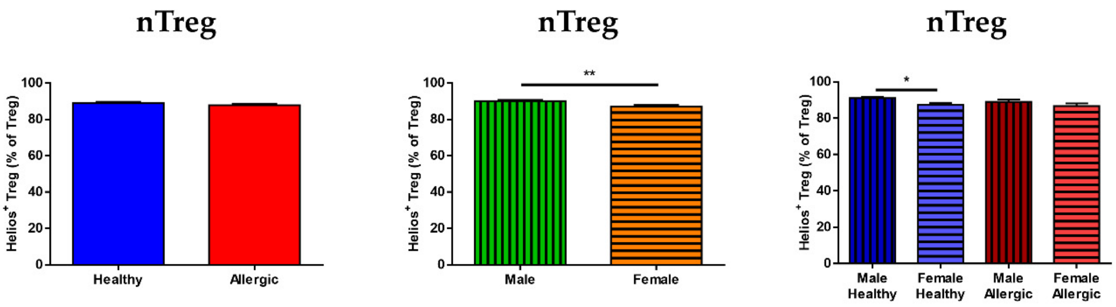

(a) (b) (c)

Supplementary figure 2. Proportions of nTreg in cord blood of male and female children of healthy and allergic mothers. Samples of cord blood (n=226) were stained and analysed by flow cytometry. (**a**) Proportion of Helios⁺ nTreg in the cord blood of children of healthy (n=118) and allergic (n=108) mothers. (**b**) Proportion of Helios⁺ nTreg in the cord blood of male (n=104) and female (n=122) newborns. * p=0.0099 (**c**) Proportion of Helios⁺ nTreg in the cord blood of newborns divided according to sex and maternal allergy status: male children of healthy mothers (n=53), female children of healthy mothers (n=65), male children of allergic mothers (n=51) and female children of allergic mothers (n=57). * p=0.0123

Supplementary figure 3

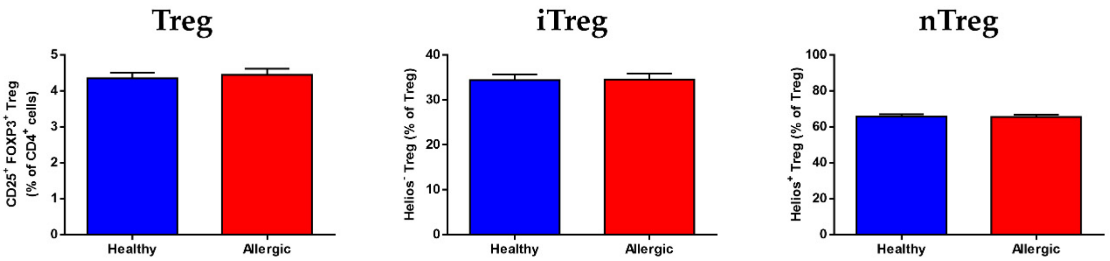

(a) (b) (c)

Supplementary figure 3. Proportions of total Treg population and iTreg and nTreg subpopulations in peripheral blood of healthy and allergic mothers. Samples of peripheral blood (n=167 total) of healthy (n=82) and allergic (n=85) mothers were stained and analysed by flow cytometry. (**a**) Proportion of CD25⁺FOXP3⁺ Treg in the CD4⁺ T-cell population of maternal peripheral blood. (**b**) Proportion of Helios⁻ iTreg in the maternal peripheral blood Treg population. (**c**) Proportion of Helios⁺ nTreg in the maternal peripheral blood Treg population.

Supplementary figure 4

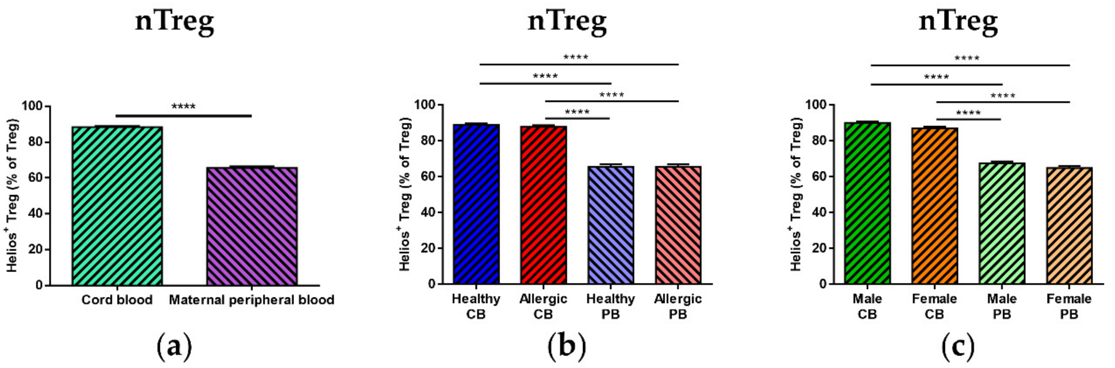

Supplementary figure 4. Comparison of nTreg between cord blood and peripheral blood of healthy and allergic mothers. Samples of cord blood (n=226) and maternal peripheral blood (n=167) were stained and analysed by flow cytometry. (**a**) Proportion of Helios$^+$ nTreg in total Treg population. **** p<0.0001 (**b**) Proportion of Helios$^+$ nTreg in total Treg in cord blood of children of healthy (n=118) and allergic (n=108) mothers and maternal peripheral blood of healthy (n=82) and allergic (n=85) mothers. **** p<0.0001 (**c**) Proportion of Helios$^+$ nTreg in total Treg in cord blood and maternal peripheral blood samples divided according to the newborns' sex: cord blood of male children (n=104), cord blood of female children (n=122), peripheral blood of mothers of male children (n=76) and peripheral blood of mothers of female children (n=86). **** p<0.0001

Supplementary figure 5

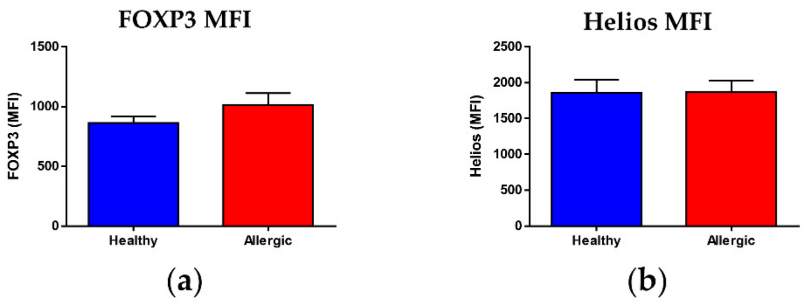

Supplementary figure 5. Median of fluorescence intensity of transcription factors FOXP3 and Helios in maternal peripheral blood Treg. Samples of peripheral blood of healthy (n=82) and allergic (n=85) mothers were stained and analysed by flow cytometry. (**a**) Median of fluorescence intensity (MFI) of FOXP3 in CD25$^+$FOXP3$^+$ Treg. (**b**) Median of fluorescence intensity (MFI) of Helios in CD25$^+$FOXP3$^+$ Treg.

Supplementary figure 6

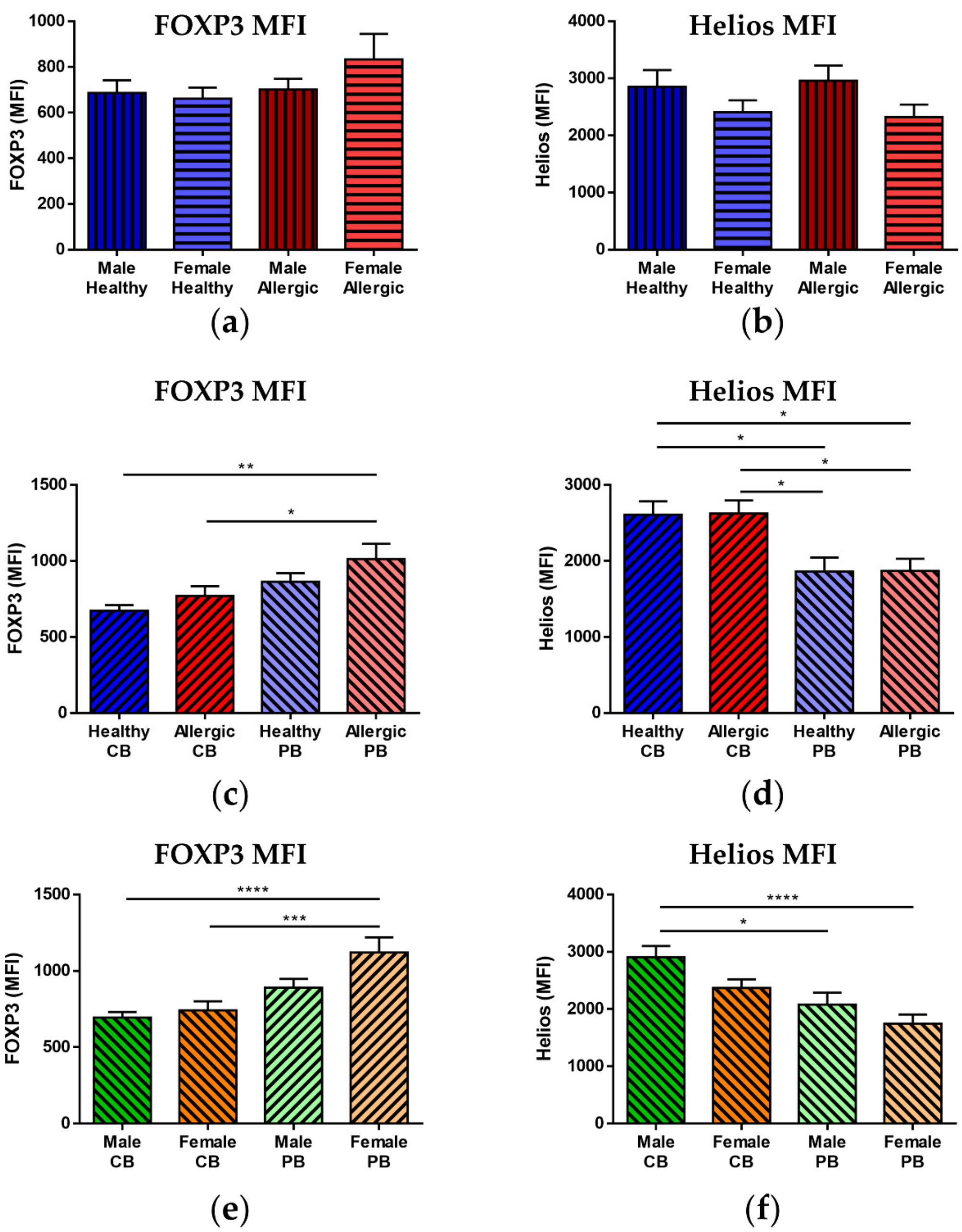

Supplementary figure 6. Median of fluorescence intensity of FOXP3 and Helios in cord blood and maternal peripheral blood analysed according to maternal allergic status and newborn sex. Samples of cord blood (n=226) and maternal peripheral blood (n=167) were stained and analysed by flow cytometry. (**a, b**) Flow cytometry analysis of cord blood samples of male children of healthy mothers (n=53), female children of healthy mothers (n=65), male children of allergic mothers (n=51) and female children of allergic mothers (n=57). (**a**) Median of fluorescence intensity (MFI) of FOXP3 in $CD25^+FOXP3^+$ Treg. (**b**) MFI of Helios in $CD25^+FOXP3^+$ Treg. (**c, d**) Flow cytometry analysis of cord blood samples of children of healthy (n=118) and allergic (n=108) mothers and maternal peripheral blood of healthy (n=82) and allergic (n=85) mothers. (**c**) MFI of FOXP3 in $CD25^+FOXP3^+$ Treg. ** p<0.01 * p<0.05 (**d**) MFI of Helios in $CD25^+FOXP3^+$ Treg. * p<0.05 (**e, f**) Flow cytometry analysis of cord blood samples of male (n=104) and female (n=122) newborns and peripheral blood of mothers bearing male (n=76) and female (n=86) children. (**e**) MFI of FOXP3 in $CD25^+FOXP3^+$ Treg. **** p<0.001 *** p<0.001 (**f**) MFI of Helios in $CD25^+FOXP3^+$ Treg. **** p<0.0001 * p<0.05

Supplementary figure 7

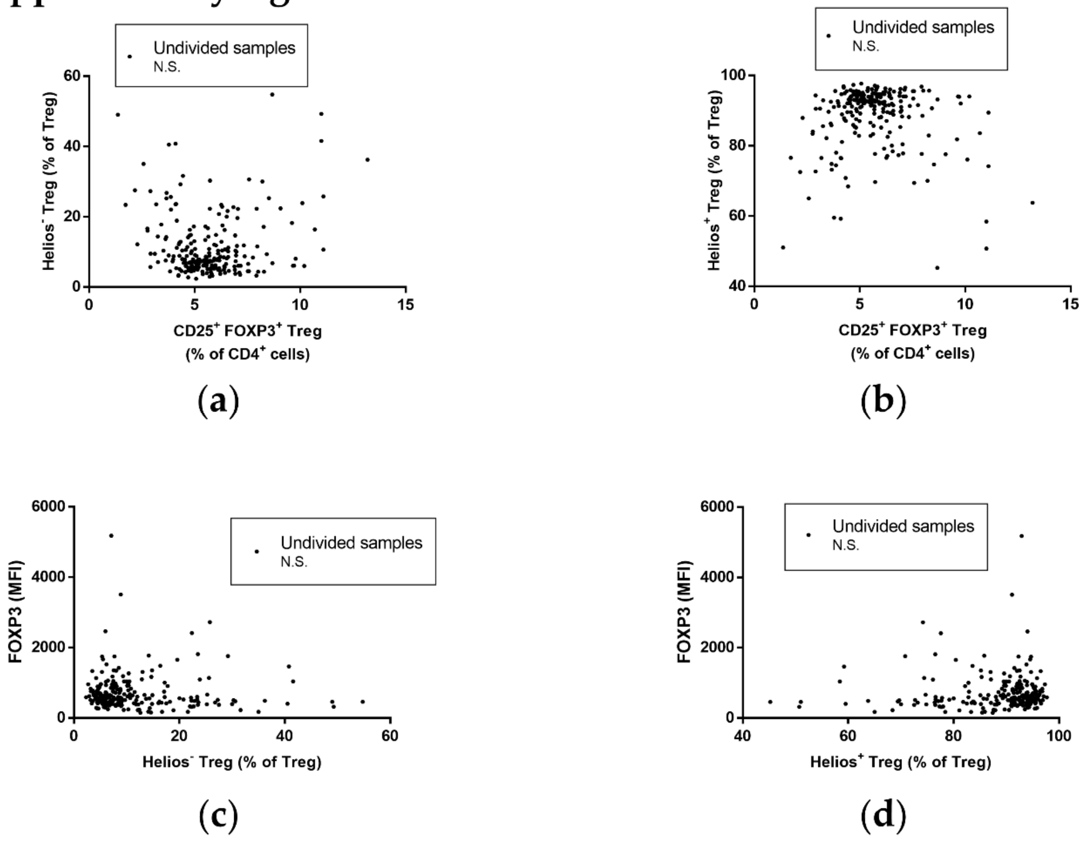

Supplementary figure 7. Correlation of selected immunoregulatory parameters in cord blood of children. Characteristics of Treg cells in cord blood (n=226) were measured using flow cytometry and correlated, utilizing Pearson's correlation coefficient. (**a**) Correlation between CD25$^+$FOXP3$^+$ Treg and Helios$^-$ iTreg in cord blood. (**b**) Correlation between CD25$^+$FOXP3$^+$ Treg and Helios$^+$ nTreg in cord blood. (**c**) Correlation between Helios$^-$ iTreg and median of fluorescence intensity (MFI) of FOXP3 in cord blood CD25$^+$FOXP3$^+$ Treg. (**d**) Correlation between Helios$^+$ nTreg and MFI of FOXP3 in cord blood CD25$^+$FOXP3$^+$ Treg. N.S., Not significant.

Supplementary figure 8

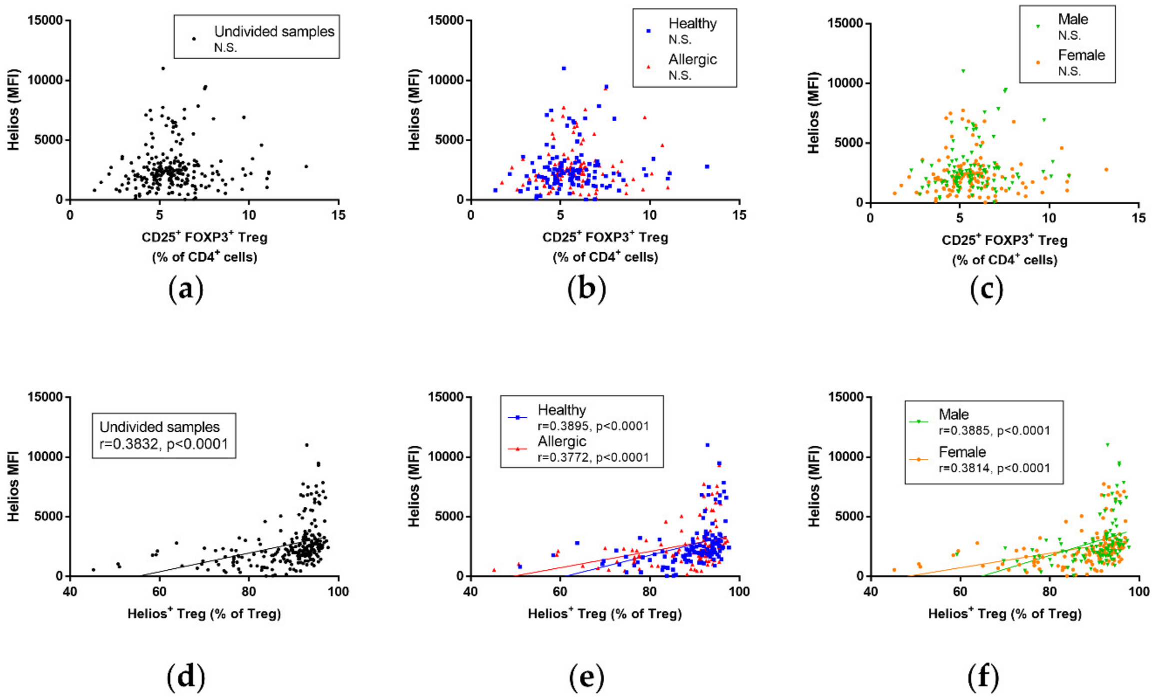

Supplementary figure 8. Correlation of selected immunoregulatory parameters in cord blood of children. Characteristics of Treg cells in cord blood of male (n=104) and female (n=122) children of healthy (n=118) and allergic (n=108) mothers were measured using flow cytometry and correlated, utilizing Pearson's correlation coefficient. (**a**-**c**) Correlation between $CD25^{+}FOXP3^{+}$ Treg and Helios^{+} nTreg in cord blood. (**a**) Correlation between $CD25^{+}FOXP3^{+}$ Treg and Helios^{-} iTreg in total cord blood samples. (**b**) Correlation between $CD25^{+}FOXP3^{+}$ Treg and Helios^{-} iTreg in cord blood of children of healthy and allergic mothers. (**c**) Correlation between $CD25^{+}FOXP3^{+}$ Treg and Helios^{-} iTreg in cord blood of male and female children. (**d**-**f**) Correlation between Helios^{+} nTreg and median of fluorescence intensity (MFI) of Helios in cord blood $CD25^{+}FOXP3^{+}$ Treg. (**d**) Correlation between Helios^{+} nTreg and MFI of Helios in cord blood $CD25^{+}FOXP3^{+}$ Treg in total cord blood samples. r=0.3832, p<0.0001 (**e**) Correlation between Helios^{+} nTreg and MFI of Helios in cord blood $CD25^{+}FOXP3^{+}$ Treg of children of healthy and allergic mothers. r=0.3985, p<0.0001 for healthy mothers, r=0.3772, p<0.0001 for allergic mothers. (**f**) Correlation between Helios^{+} nTreg and MFI of Helios in cord blood $CD25^{+}FOXP3^{+}$ Treg of male and female children. r=0.3885, p<0.0001 for male, r=0.3814, p<0.0001 for female. N.S., Not significant.

Supplementary figure 9

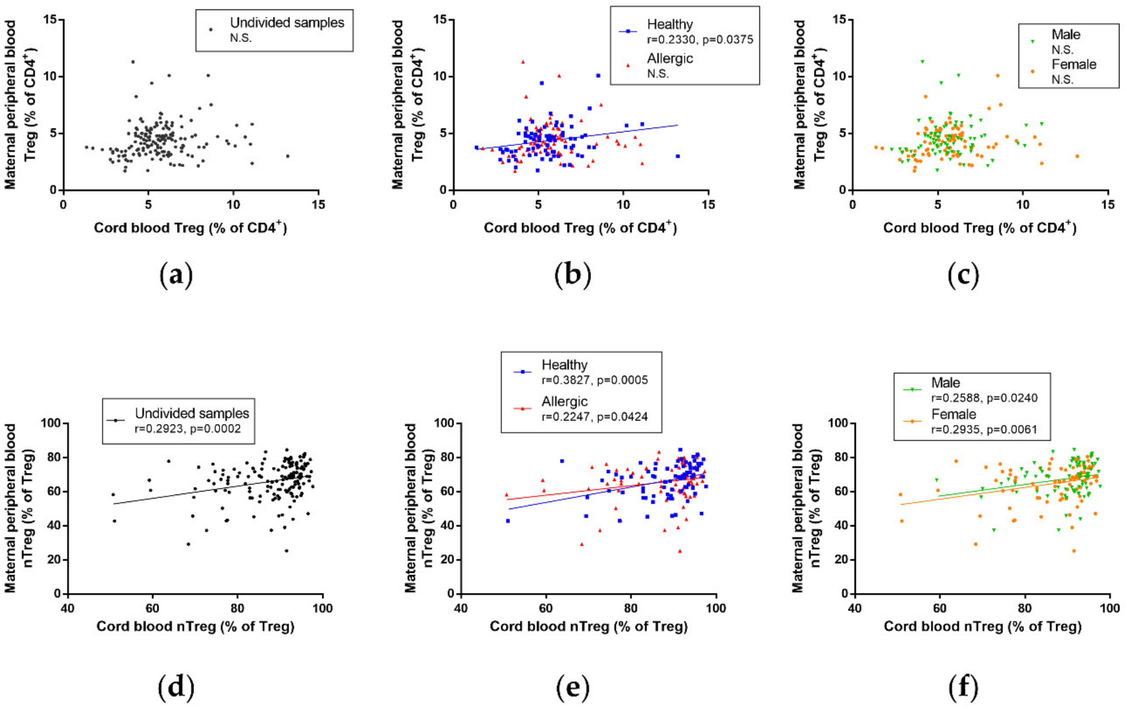

Supplementary figure 9. Correlation of Treg subpopulations between cord blood of children and maternal peripheral blood of their mothers. Proportion characteristics of CD25+FOXP3+ Treg and nTreg cells in cord blood of male (n=76) and female (n=86) children of healthy (n=80) and allergic (n=82) mothers was measured using flow cytometry and correlated with characteristics in maternal peripheral blood, utilizing Pearson's correlation coefficient. (**a**-**c**) Correlation between CD25+FOXP3+ Treg in cord blood of male and female children of healthy and allergic children and peripheral blood of their mothers. (**a**) Correlation between CD25+FOXP3+ Treg in cord blood of children and peripheral blood of their mothers. (**b**) Correlation between CD25+FOXP3+ Treg in cord blood of children of healthy and allergic mothers and maternal peripheral blood. r=0.2330, p=0.0375 for healthy mothers. (**c**) Correlation between CD25+FOXP3+ Treg in cord blood of male and female children and peripheral blood of their mothers. (**d**-**e**) Correlation between Helios+ nTreg in cord blood of male and female children of healthy and allergic children and peripheral blood of their mothers. (**d**) Correlation between Helios+ nTreg in cord blood of children and peripheral blood of their mothers (n=162). r=0.2923, p=0.0002 (**e**) Correlation between Helios+ nTreg in cord blood of children and peripheral blood of healthy and allergic mothers. r=0.3827, p=0.0005 for healthy mothers, r=0.2247, p=0.0424 for allergic mothers. (**f**) Correlation between Helios+ Treg in cord blood of male and female children and peripheral blood of their mothers. r=0.2588, p=0.0240 for male children, r=0.2935, p=0.0061 for female children. N.S., Not significant.

Supplementary figure 10

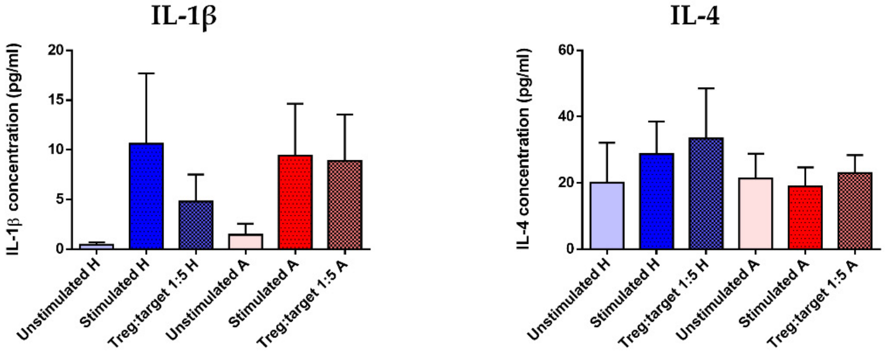

(**a**) (**b**)

Supplementary figure 10. Concentration of selected cytokines in supernatants of Treg-Tconv cocultures. Non-Treg CD4+ T-cells (Tconv) magnetically isolated from children of healthy (n=14) and allergic (n=15) mothers were cultured with IL-2, unstimulated (negative control) or stimulated with a cocktail of functional-grade anti-CD3 + anti-CD28 antibodies (positive control). Treg magnetically isolated from the same cord blood samples were added to stimulated Tconv at 1:5 Treg:Tconv ratio. Culture supernatants were collected after 72h of culture and concentrations of selected cytokines in supernatant were measured with ELISA. (**a**) Concentrations of IL-1β in the supernatant of unstimulated Tconv, stimulated Tconv and Treg:Tconv cocultures of Tconv and Treg of children of healthy and allergic mothers. (**b**) Concentrations of IL-4 in the supernatant of unstimulated Tconv, stimulated Tconv and Treg:Tconv cocultures of Tconv and Treg of children of healthy and allergic mothers.

Supplementary figure 11

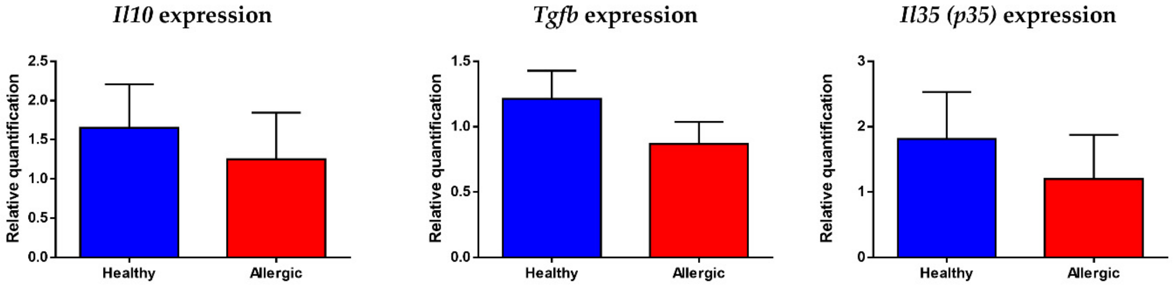

(**a**) (**b**) (**c**)

Supplementary figure 11. **Analysis of regulatory cytokine gene expression in magnetically isolated Treg.** Total RNA was obtained from Treg magnetically isolated from cord blood samples of children of healthy (n=9) and allergic (n=10) mothers. Reverse transcription was performed and mRNA content was determined using real-time quantitative PCR. (**a**) Gene expression of *Il10* in cord blood Treg of healthy and allergic mothers. (**b**) Gene expression of *Tgfb* in cord blood Treg of healthy and allergic mothers. (**c**) Gene expression of *Il35* (*p35* subunit) in cord blood Treg of healthy and allergic mothers.

Supplementary figure 12

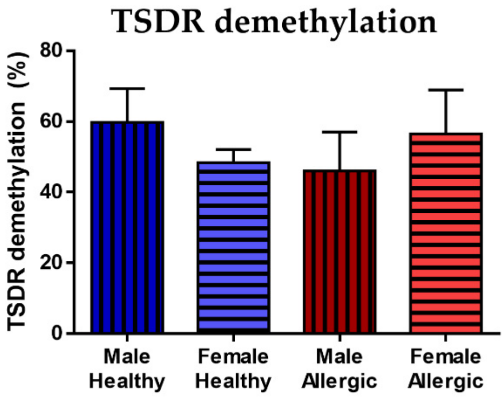

(a)

Supplementary figure 12. Epigenetic analysis of demethylation of TSDR locus of FOXP3 promoter. Genomic DNA was obtained from Treg magnetically isolated from cord blood samples (n=27). Bisulphite conversion of DNA was performed and methylation status of converted DNA samples was measured using high-resolution melting PCR analysis. (**a**) Percentage of TSDR demethylation of DNA isolated from male children of healthy mothers (9), female children of healthy mothers (n=4), male children of allergic mothers (n=9) and female children of allergic mothers (n=5).

Supplementary table 1. Correlation of Treg characteristics in cord blood.

Correlated factors	Undivided samples	Healthy mothers	Allergic mothers	Male children	Female children
**total Treg × iTreg**	*N.S.* [1]	*N.S.*	*N.S.*	*N.S.*	*N.S.*
**total Treg × nTreg**	*N.S.*	*N.S.*	*N.S.*	*N.S.*	*N.S.*
**FOXP3 MFI** [2] **× total Treg**	*r= 0.1400 p= 0.0354*	*r= 0.2271 p= 0.0134*	*N.S.*	*r= 0.2762 p= 0.0045*	*N.S.*
**FOXP3 MFI × iTreg**	*N.S.*	*N.S.*	*N.S.*	*N.S.*	*N.S.*
**FOXP3 MFI × iTreg**	*N.S.*	*N.S.*	*N.S.*	*N.S.*	*N.S.*
**Helios MFI × total Treg**	*N.S.*	*N.S.*	*N.S.*	*N.S.*	*N.S.*
**Helios MFI × iTreg**	*r= -0.3833 p <0.0001*	*r= -0.3985 p <0.0001*	*r= -0.3774 p <0.0001*	*r= -0.3888 p <0.0001*	*r= -0.3814 p <0.0001*
**Helios MFI × nTreg**	*r= 0.3832 p <0.0001*	*r= 0.3895 p <0.0001*	*r= 0.3772 p <0.0001*	*r= 0.3885 p <0.0001*	*r= 0.3814 p <0.0001*

[1] Not significant. [2] median of fluorescence intensity. r – Pearson's correlation coefficient

Supplementary table 2. Correlation of Treg characteristics between cord blood and maternal peripheral blood.

Correlated factors	Undivided samples	Healthy mothers	Allergic mothers	Male children	Female children
**total Treg CB** [1] **× PB** [2]	*N.S.* [3]	*r= 0.2330 p= 0.0375*	*N.S.*	*N.S.*	*N.S.*
**iTreg CB × PB**	*r= 0.2923 p= 0.0002*	*r= 0.3825 p= 0.0005*	*r= 0.2249 p= 0.0423*	*r= 0.2591 p= 0.0238*	*r= 0.2933 p= 0.0061*
**nTreg CB × PB**	*r= 0.2923 p= 0.0002*	*r= 0.3827 p= 0.0005*	*r= 0.2247 p= 0.0424*	*r= 0.2588 p= 0.0240*	*r= 0.2935 p= 0.0061*

[1] Cord blood [2] Maternal peripheral blood [3] Not significant. r – Pearson's correlation coefficient
